# Mesenchymal stem cell therapy as adjunctive treatment in pediatric patients with severe acquired brain injury: a single-center retrospective case series

**DOI:** 10.3389/fped.2026.1859885

**Published:** 2026-07-14

**Authors:** Hacer Uçmak, Merve Havan, Anar Gurbanov, Burak Balaban, Fevzi Kahveci, Hasan Özen, Edin Botan, Emrah Gün, Ayşen Durak Aslan, Eda Eyduran, Ömer Bektaş, Ayşe Eser Elçin, Tanıl Kendirli

**Affiliations:** 1 Department of Pediatrics, Division of Pediatric Intensive Care, Ankara University, Ankara, Türkiye; 2 Department of Pediatrics, Division of Pediatric Neurology, Ankara University, Ankara, Türkiye; 3 Stem Cell Institute, Tissue and Cell Production Center, Ankara University, Ankara, Türkiye; 4 Department of Pediatric Critical Care, Ankara University School of Medicine, Ankara, Türkiye

**Keywords:** hypoxic-ischemic encephalopathy, mesenchymal stem cells, pediatric brain injury, pediatric cerebral performance category, pediatric intensive care

## Abstract

**Objective:**

Severe acquired brain injury in critically ill children—most commonly hypoxic-ischemic in origin—frequently results in long-term neurological disability. Conventional pediatric intensive care management focuses on preventing secondary brain injury, but therapeutic options targeting neurorestoration remain limited. Mesenchymal stem cells (MSCs), through paracrine, immunomodulatory, anti-inflammatory, and trophic effects, have been proposed as an experimental adjunctive therapy. We aimed to describe the clinical course and short-term neurological outcomes of pediatric patients who received MSC therapy as an adjunct to conventional intensive care in our pediatric intensive care unit (PICU).

**Patients and methods:**

This single-center retrospective case series included pediatric patients who received adjunctive MSC therapy in our PICU between January 2019 and August 2023. Demographic and clinical characteristics, conventional treatments received, MSC administration parameters, baseline and follow-up neuroimaging when available, and neurological outcomes were extracted from hospital records. Allogeneic bone marrow- or umbilical cord blood-derived MSCs were administered at a dose of 1–2 × 10^6^ cells/kg per session over 3–5 sessions, intravenously and/or intrathecally. Neurological outcome was assessed using the Glasgow Coma Scale (GCS) and the Pediatric Cerebral Performance Category (PCPC) at baseline, 1 month, and 6 months after MSC therapy.

**Results:**

Six patients (5 males, 1 female; median age 65.5 months, range 2–204) received adjunctive MSC therapy. Five had hypoxic brain injury and one had penetrating traumatic brain injury (gunshot wound). All patients had a baseline PCPC of 5 (coma/vegetative state). Median GCS improved from 4 (range 3–5) before MSC therapy to 11 (range 8–15) after the treatment course. PCPC decreased over time (median 5 at baseline, 4 at 1 month, 3 at 6 months). No procedure-related adverse events were observed during MSC administration or during the 6-month follow-up. All six patients survived.

**Conclusion:**

In this small retrospective case series, adjunctive MSC therapy was feasible and well tolerated in pediatric patients with severe acquired brain injury, and neurological improvement was observed during follow-up. Because of the small sample size, lack of a control group, heterogeneous etiologies and protocols, and concomitant conventional care and rehabilitation, neurological improvement cannot be attributed to MSC therapy alone. These observations are hypothesis-generating; controlled prospective studies are required to establish efficacy.

## Introduction

Severe acquired brain injury is a leading cause of long-term neurological disability and mortality in critically ill children. In the pediatric intensive care unit (PICU), the most frequent etiologies include hypoxic-ischemic injury after cardiac or respiratory arrest, traumatic brain injury (TBI), central nervous system infections, and metabolic insults (1–3). Despite advances in neurocritical care, conventional management remains largely supportive: it aims to prevent secondary brain injury through mechanical ventilation, hemodynamic optimization, control of intracranial pressure (ICP), seizure control, normothermia, glycemic and electrolyte management, and early initiation of rehabilitation. While these measures reduce secondary damage, they do not directly promote neural repair, and many survivors are left with persistent motor, cognitive, and behavioral deficits. This therapeutic gap has motivated the search for adjunctive treatments capable of supporting neurorestoration (4).

Mesenchymal stem cells (MSCs) are multipotent stromal cells that can be isolated from various tissues, most commonly bone marrow, adipose tissue, and umbilical cord (Wharton's jelly and umbilical cord blood) (5–7). According to the International Society for Cellular Therapy (ISCT) minimal criteria, MSCs are defined by plastic adherence, expression of CD73, CD90, and CD105, lack of expression of hematopoietic markers (CD34, CD45, CD14, CD11b, CD19, HLA-DR), and the capacity for tri-lineage mesenchymal differentiation (8). It is important to distinguish MSCs from bone marrow mononuclear cells (BM-MNCs) and umbilical cord blood mononuclear cells (UCB-MNCs), which are heterogeneous mixtures of hematopoietic and stromal lineage cells and are biologically distinct from purified, characterized MSC products (9). The clinical literature in pediatric brain injury includes reports using each of these cell products, and they should not be considered interchangeable when interpreting outcomes (10–12).

The therapeutic justification for MSC application in acquired brain injury is grounded in their paracrine and immunomodulatory contributions rather than in direct cell replacement strategies (1, 2). MSCs—particularly through secretion of trophic factors, anti-inflammatory cytokines, and extracellular vesicles/exosomes—have been shown in preclinical models to attenuate microglial activation, promote angiogenesis, support endogenous neurogenesis, restore blood-brain barrier integrity, and reduce reactive gliosis (13–17). Definitive evidence for direct trans-differentiation of MSCs into functional neurons *in vivo* remains limited; current data favor paracrine and immunomodulatory mechanisms as the dominant mode of action (14, 17).

Clinical experience with cell therapy in pediatric acquired brain injury remains limited and heterogeneous. In TBI, autologous BM-MNC infusion has been reported as feasible and safe, with potential reductions in therapeutic intensity and early ICU resource use (11, 12, 18). In hypoxic-ischemic encephalopathy and cerebral palsy, intrathecal UC-MSCs and allogeneic umbilical cord blood products have been associated with motor and functional gains in some studies, although results are variable and the underlying cell products differ substantially (19–23). Phase I trials of MSC therapy in pediatric severe TBI have reported safety and preliminary functional improvement (10, 11, 24). Despite this growing body of preliminary evidence, robust randomized controlled data are lacking, and most published cohorts are small, heterogeneous, and uncontrolled (4, 10).

In this context, we aimed to describe our single-center clinical experience with adjunctive MSC therapy in critically ill pediatric patients with severe acquired brain injury. We report this experience as a retrospective, hypothesis-generating case series intended to add to the descriptive clinical literature, not to establish efficacy.

## Patients and methods

This is a single-center retrospective case series conducted at the Pediatric Intensive Care Unit (PICU) of Ankara University Faculty of Medicine, a tertiary academic referral center serving patients from across Türkiye. The study was approved by the Ethics Committee of Ankara University (decision number 2025/336). Written informed consent for the MSC treatment was obtained from the parents or legal guardians of all patients prior to administration, as part of routine clinical care. The study was conducted in accordance with the principles of the Declaration of Helsinki.

### Patient selection

We retrospectively reviewed pediatric patients who received adjunctive MSC therapy in our PICU between January 2019 and August 2023. Eligibility for MSC therapy in clinical practice required all of the following: (i) a diagnosis of severe acquired brain injury (traumatic or non-traumatic) within approximately one month of the index event; (ii) persistent severely impaired consciousness despite optimal conventional intensive care, defined as a Glasgow Coma Scale (GCS) score of 8 or less and a Pediatric Cerebral Performance Category (PCPC) score consistent with coma/vegetative state; (iii) clinical stability sufficient to undergo cell therapy in the subacute or chronic phase; (iv) absence of contraindications to the chosen route of administration (notably, no signs of significantly elevated ICP, herniation, or active intracranial hemorrhage for the intrathecal route); and (v) a multidisciplinary decision to proceed taken jointly by pediatric intensive care and pediatric neurology specialists, followed by detailed family counseling and informed consent.

Because MSC therapy is not reimbursed by the Turkish national health insurance system for any pediatric indication and is regarded as an adjunctive, non-standard treatment rather than a primary therapy, families were thoroughly counseled regarding its experimental nature, potential procedural risks, the theoretical concern of tumorigenesis, the absence of guaranteed benefit, and the out-of-pocket cost. Treatment was administered only after the family provided informed consent and was able to access the therapy. Approval from the Türkiye Ministry of Health Tissue and Organ Transplantation Coordination Center was obtained for each patient prior to administration. The selection bias inherent in this access pathway is acknowledged in the Limitations section.

### Conventional treatment

All patients received standardized conventional pediatric neurocritical care prior to and concurrent with MSC therapy, in accordance with current institutional and international PICU practice (1). Components included mechanical ventilation with protective ventilation strategies as clinically indicated; hemodynamic management aimed at maintaining age-appropriate normotension and adequate cerebral perfusion; active maintenance of normothermia and avoidance of hyperthermia (therapeutic hypothermia was not applied in this cohort, as targeted temperature management for brain protection in our institutional pediatric practice is reserved for neonatal hypoxic-ischemic encephalopathy and was not indicated for the patients in this series); ICP management guided by clinical and neuroimaging assessment, given that invasive ICP monitoring is not routinely available in pediatric practice in our setting—when cerebral edema or intracranial hypertension was suspected, hyperosmolar therapy with hypertonic saline was the agent of choice (mannitol is not used in our pediatric protocol), together with adequate sedation and analgesia; antiepileptic prophylaxis initiated in all patients with continuous bedside electroencephalographic (cEEG) monitoring to detect and guide management of subclinical and clinical seizure activity; maintenance of normal serum electrolytes, hemoglobin, and platelet counts to optimize cerebral oxygen delivery and perfusion; empirical and targeted antimicrobial therapy when infection was suspected or confirmed; early enteral nutritional support whenever feasible; and early initiation of physical therapy and rehabilitation, including passive range-of-motion, positioning, and pulmonary physiotherapy from the early stable phase onward.

### MSC product, preparation, and characterization

Allogeneic MSC products were prepared at the Tissue and Cell Production Center of the Ankara University Stem Cell Institute, a Ministry of Health-licensed facility. MSCs were derived from human bone marrow or human umbilical cord blood depending on availability and individual treatment plans (6, 25). All MSC products were prepared, expanded, and characterized in accordance with the ISCT minimal criteria for defining multipotent mesenchymal stromal cells, including plastic adherence, expression of CD73, CD90, and CD105 (≥95%), absence of expression of CD34, CD45, CD14, CD11b, CD19, and HLA-DR (≤2%), and demonstrated tri-lineage mesenchymal differentiation capacity (7, 8). Sterility, mycoplasma, endotoxin, and viability testing were performed prior to release of each product, in line with national tissue and cell production standards.

### MSC administration protocol

Patients received 3–5 sessions of allogeneic MSCs at a dose of 1–2 × 10^6^ cells per kilogram of body weight per session. Doses were administered intravenously and/or intrathecally; the choice of route was individualized based on clinical contraindications. The intravenous (IV) route was preferred when intrathecal administration was contraindicated, in particular when there was significant intracranial hemorrhage, suspected elevated ICP, cerebral edema, or radiographic evidence of mass effect (26). The intrathecal (IT) route was selected when no contraindications were present, with the goal of direct delivery to the cerebrospinal fluid compartment (22). In selected patients, IV and IT routes were combined in different sessions to leverage both systemic and direct central nervous system exposure.

The interval between sessions ranged from approximately 10 days to 2 weeks and was determined by the availability of each MSC product, rather than by a fixed pre-planned schedule. This reflects the real-world clinical context of MSC product preparation and delivery in our setting; a standardized fixed-interval protocol was not used (27). All administrations were performed in a monitored setting with continuous vital sign and neurological surveillance.

### Outcome measures

The primary clinical outcome measures were the age-appropriate Glasgow Coma Scale (GCS)—the pediatric GCS for children younger than 5 years and the standard GCS for older patients—and the Pediatric Cerebral Performance Category (PCPC). The PCPC is a six-level ordinal scale (1 = normal; 2 = mild disability; 3 = moderate disability; 4 = severe disability; 5 = coma/vegetative state; 6 = brain death). PCPC was recorded at baseline (before MSC therapy), at 1 month, and at 6 months after the MSC therapy course. GCS was recorded before MSC therapy and at the end of the 6-month follow-up. Because most patients were intubated, mechanically ventilated, and receiving continuous sedation, GCS assessment was performed during planned interruptions of sedation (sedation holds) whenever clinically safe. The verbal component was modified or marked as non-testable in patients who were intubated, tracheostomy-dependent, or otherwise unable to vocalize, in accordance with standard practice. Neurodevelopmental status was interpreted relative to the patient's age. These factors are acknowledged as potential confounders in the interpretation of GCS changes in this cohort. Severity of critical illness at PICU admission was characterized using the Pediatric Risk of Mortality III (PRISM III) and Pediatric Logistic Organ Dysfunction 2 (PELOD-2) scores. Other recorded variables included demographic data, comorbidities, indication for MSC therapy, ventilator support, MSC source, route, dose per session, total number of sessions, length of PICU and hospital stay, and outcome.

### Neuroimaging and EEG

Because our PICU is a tertiary referral center, most patients were transferred from other healthcare facilities at variable stages of their illness, and after PICU stabilization were referred back to pediatric neurology services in their home provinces for long-term follow-up. Baseline and follow-up neuroimaging studies [computed tomography (CT) and magnetic resonance imaging (MRI)] and electroencephalography were therefore performed across multiple institutions using non-standardized protocols, varying equipment, and at variable time points relative to the index injury. Available imaging studies and reports were reviewed when accessible. A systematic, longitudinal quantitative imaging analysis was not feasible; however, qualitative baseline and follow-up imaging features are summarized in the Results.

### Safety monitoring

Safety was prospectively assessed in clinical practice and reviewed retrospectively for the purposes of this report. Procedure-related adverse events were systematically monitored during each MSC infusion and for at least 24 h afterward, and included: fever, infusion-related reactions, allergic/anaphylactic reactions, hemodynamic instability, respiratory deterioration, and pulmonary embolism (for IV administration); fever, headache, signs of raised ICP, nausea/vomiting, neurological deterioration, and CSF infection or chemical meningitis (for IT administration). Longer-term safety surveillance during the 6-month follow-up included new neurological deficits, increased seizure burden, and clinical or radiographic signs suggestive of tumorigenesis.

### Statistical analysis

Given the descriptive nature of this case series and the very small sample size (*n* = 6), analyses were primarily descriptive. Continuous variables are reported as median (interquartile range, IQR) and minimum–maximum, and categorical variables as counts and percentages. The Wilcoxon signed-rank test was used to compare paired pre- and post-treatment GCS values. The Friedman test was used to compare paired PCPC values across three time points (baseline, 1 month, 6 months). PCPC, an ordinal scale, is reported as median (range/IQR) at each time point. Statistical significance was set at *p* < 0.05; however, given the small sample size and uncontrolled design, *p*-values should be interpreted as describing within-cohort change over time and cannot be interpreted as evidence that observed improvement was caused by MSC therapy. Analyses were performed using IBM SPSS Statistics version 21.

## Results

### Patient characteristics

Six pediatric patients (5 males, 1 female) received adjunctive MSC therapy during the study period. Median age at MSC therapy was 65.5 months (range 2–204), and median body weight was 27.0 kg (range: 5–56). Comorbidities were present in 5 of 6 patients and included neonatal hemorrhagic disease, congenital laryngeal atresia requiring permanent tracheostomy for airway maintenance, non-compaction cardiomyopathy, primary immunodeficiency, and a life-threatening arrhythmia. The indication for MSC therapy was hypoxic-ischemic brain injury following cardiac or respiratory arrest in five patients and penetrating traumatic brain injury from a gunshot wound in one patient. Median PRISM III score at PICU admission was 9.5 (range: 4–16), and median PELOD-2 score was 21 (range: 21–27). Patient-level demographic, clinical, and treatment data are presented in Table 1.

**Table 1:** Demographic, clinical, and treatment characteristics of the six pediatric patients who received adjunctive MSC therapy.

Variable	P1	P2	P3	P4	P5	P6
Sex	M	F	M	M	M	M
Age (months)	2	10	121	9	204	143
Body weight (kg)	5	9	45	9	56	50
Comorbidity	Neonatal hemorrhagic disease	Laryngeal atresia, tracheostomy	Non-compaction cardiomyopathy	Primary immunodeficiency	Arrhythmia	None
PICU admission reason	Respiratory arrest	Respiratory arrest	Cardiac arrest	Cardiac arrest + MCA infarction	Cardiac arrest	Gunshot wound (penetrating TBI)
PRISM III	10	5	9	16	15	4
PELOD-2	22	21	21	21	27	21
Indication for MSC	Hypoxic	Hypoxic	Hypoxic	Hypoxic	Hypoxic	TBI
Pre-MSC GCS	4	3	3	5	5	3
Pre-MSC PCPC	5	5	5	5	5	5
Pre-MSC mech. ventilation	Yes	Yes	Yes	Yes	Yes	Yes
Pre-MSC MV duration (days)	49	305	31	20	21	20
MSC source	Allogeneic BM	Allogeneic BM	Allogeneic BM	Allogeneic BM	Allogeneic UCB	Allogeneic UCB
Dose per session ( × 10^6^ cells/kg)	1–2	1–2	1–2	1–2	1–2	1–2
Number of sessions	3	5	4	3	3	3
Route	IV	IT	IT + IV	IV	IT + IV	IT + IV
Post-MSC GCS (6 mo)	10	9	8	11	15	15
Post-MSC mech. ventilation	No	Yes (tracheostomy)	Yes (tracheostomy)	No	Yes	No
PCPC at 1 month	4	4	4	3	3	3
PCPC at 6 months	4	4	4	2	1	2
PICU stay (days)	75	83	56	37	47	67
Hospital stay (days)	111	152	96	249	88	92
Outcome at 6 mo	Survived	Survived	Survived	Survived	Survived	Survived

BM, bone marrow; F, female; GCS, Glasgow Coma Scale; IT, intrathecal; IV, intravenous; M, male; MCA, middle cerebral artery; MSC, mesenchymal stem cell; MV, mechanical ventilation; P, patient; PCPC, Pediatric Cerebral Performance Category; PELOD-2, Pediatric Logistic Organ Dysfunction 2; PICU, pediatric intensive care unit; PRISM III, Pediatric Risk of Mortality III; TBI, traumatic brain injury; UCB, umbilical cord blood. PCPC: 1 = normal, 2 = mild disability, 3 = moderate disability, 4 = severe disability, 5 = coma/vegetative state, 6 = brain death. MSC dose was 1–2 × 10^6^ cells/kg per session for all patients; the previously reported total cell counts represented patient body weight values rather than per-kilogram doses and have been corrected accordingly.

### Conventional treatment and pre-MSC course

All six patients required mechanical ventilation prior to MSC therapy, with a median pre-MSC ventilation duration of 26 days (range: 20–305). All patients received the standardized conventional neurocritical care described in the Methods, including continuous bedside EEG monitoring with antiepileptic therapy initiated upon detection of subclinical or clinical seizure activity; hemodynamic management directed at preserving adequate cerebral perfusion pressure while avoiding excessive systemic hypertension, with antihypertensive therapy administered when clinically required; sedation and analgesia provided on a clinical-need basis for pain, agitation, or patient–ventilator dyssynchrony; hyperosmolar therapy with hypertonic saline in the presence of cerebral edema; maintenance of normothermia; and early initiation of physical therapy and rehabilitation.

#### MSC therapy

MSCs were derived from allogeneic bone marrow in four patients and from allogeneic umbilical cord blood in two patients. Each patient received 3–5 sessions (median 3) at a dose of 1–2 × 10^6^ cells/kg per session. The route of administration was intrathecal (IT) only in one patient, intravenous (IV) only in two patients, and combined IT + IV in three patients. The IV route was used in patients with significant intracranial hemorrhage, mass effect, or clinical signs suggestive of raised intracranial pressure, whereas the IT route was reserved for patients without these contraindications.

#### Neurological outcomes

The median GCS improved from 4 (range 3–5; IQR 3–5) before MSC therapy to 11 (range 8–15; IQR 9–14) after the 6-month follow-up; this within-cohort change was statistically significant (Wilcoxon signed-rank *Z* = −2.226; *p* = 0.026). The PCPC was 5 (coma/vegetative state) at baseline in all six patients. At 1 month, PCPC had decreased in all patients (three patients to PCPC 4 and three to PCPC 3). At 6 months, PCPC decreased further: one patient improved to PCPC 1 (normal), two patients to PCPC 2 (mild disability), and three patients remained at PCPC 4 (severe disability). The median PCPC was 5 at baseline, 4 at 1 month, and 3 at 6 months. The Friedman test for repeated measures across the three time points yielded *χ*^2^ = 11.143, *p* = 0.004. As emphasized in the Methods, this *p*-value reflects within-cohort change over time and does not establish a causal effect of MSC therapy. PCPC values at each time point are summarized in Table 2.

**Table 2:** Summary statistics for clinical variables and PCPC at each follow-up time point (*n* = 6).

Variable	Median (IQR)	Min	Max
Age (months)	65.5 (9–138)	2	204
Body weight (kg)	27.0 (9–49)	5	56
PRISM III	9.5 (6–14)	4	16
PELOD-2	21 (21–22)	21	27
MV duration before MSC (days)	26 (20–44)	20	305
Number of MSC sessions	3 (3–4)	3	5
PICU stay (days)	61.5 (49–73)	37	83
Hospital stay (days)	103.5 (93–142)	88	249
GCS—pre-MSC	4 (3–5)	3	5
GCS—post-MSC (6 mo)	11 (9–14)	8	15
PCPC—baseline	5 (5–5)	5	5
PCPC—1 month	4 (3–4)	3	4
PCPC—6 months	3 (2–4)	1	4

GCS, Glasgow Coma Scale; IQR, interquartile range; MSC, mesenchymal stem cell; MV, mechanical ventilation; PCPC, Pediatric Cerebral Performance Category; PELOD-2, Pediatric Logistic Organ Dysfunction 2; PICU, pediatric intensive care unit; PRISM III, Pediatric Risk of Mortality III. PCPC is reported as median (IQR) at each time point because of its ordinal nature; the Friedman test for repeated measures across the three time points yielded *χ*^2^ = 11.143, *p* = 0.004. The Wilcoxon signed-rank test for paired pre- vs. post-MSC GCS yielded *Z* = −2.226, *p* = 0.026. Both *p*-values describe within-cohort change over time and do not establish a causal effect of MSC therapy.

Three patients were successfully weaned from mechanical ventilation following MSC therapy, while three remained on respiratory support (two via tracheostomy). All six patients survived, with median PICU and hospital stays of 61.5 days (range 37–83) and 103.5 days (range 88–249), respectively.

#### Imaging findings

Baseline and follow-up neuroimaging was heterogeneous in modality, timing, and source institution, as detailed in the Methods. The dominant imaging patterns were as follows.

In Patient 1 (2-month-old infant with neonatal hemorrhagic disease and respiratory arrest), baseline CT and MRI demonstrated acute right convexity subdural hemorrhage with up to 1 cm thickness extending to the right Sylvian fissure, diffuse cerebral and cerebellar edema with sulcal effacement, midline shift, tonsillar herniation, and right frontoparietal craniectomy defects with parenchymal protrusion. Follow-up MRI and CT showed extensive bilateral cerebral encephalomalacia, cerebellar atrophy, ventricular enlargement, and persistent craniectomy defects, consistent with severe global hypoxic-ischemic and post-hemorrhagic injury.

In Patient 2 (10-month-old with laryngeal atresia and chronic tracheostomy), neck and thoracic imaging confirmed laryngeal atresia and tracheal/bronchial irregularity related to long-standing tracheostomy; brain imaging was consistent with chronic post-hypoxic changes.

In Patient 3 (10-year-old with non-compaction cardiomyopathy), MRI demonstrated a classic deep hypoxic-ischemic injury pattern, with bilateral perirolandic and parieto-occipital cortical-subcortical volume loss, basal ganglia involvement (cavitary encephalomalacia in globus pallidus on follow-up), ulegyric appearance in parieto-occipital cortex, FLAIR hyperintensity in the corticospinal tracts and periaqueductal region, and progressive cerebral and cerebellar atrophy with ventricular enlargement on follow-up imaging (3).

In Patient 4 [9-month-old with primary immunodeficiency, cardiac arrest with concurrent middle cerebral artery (MCA) infarction], follow-up MRI revealed cystic encephalomalacia and gliosis in the left MCA territory with involvement of the left thalamus and lentiform nucleus, additional patchy white matter signal changes in the right hemisphere, and crossed cerebellar diaschisis—consistent with combined hypoxic-ischemic and large-vessel ischemic injury (28).

In Patient 5 (17-year-old adolescent with arrhythmia and cardiac arrest), baseline CT showed hypodensity in deep white matter at the centrum semiovale and bilateral perirolandic cortex, and baseline contrast-enhanced MRI demonstrated bilateral symmetric cortical diffusion restriction with corresponding T2/FLAIR hyperintensity in the bilateral globus pallidus, thalami, splenium of the corpus callosum, and midbrain corticospinal tracts, consistent with classic hypoxic-ischemic injury and post-anoxic leukoencephalopathy.

In Patient 6 (12-year-old with penetrating TBI from a gunshot wound, no comorbidity), baseline CT and MRI demonstrated a right parietal entry and left parietal exit defect with bone fragments along the bullet tract, dense subgaleal hemorrhage, parenchymal hemorrhage along the trajectory with surrounding edema, and dense subarachnoid and thin subdural hemorrhage. Follow-up CT approximately 20 months later showed cystic encephalomalacia along the bullet tract in both cerebral hemispheres, cerebral atrophy, and asymmetric ventricular enlargement, with no evidence of new ischemic or hemorrhagic injury.

#### Safety

No procedure-related adverse events were observed during MSC administration or throughout the 6-month follow-up. Specifically, no fever, infusion-related or allergic reaction, hemodynamic instability, respiratory deterioration, headache, vomiting, signs of raised ICP, neurological deterioration, CSF infection, increased seizure burden, or clinical or radiographic signs of tumorigenesis were detected during structured monitoring.

## Discussion

Our findings are consistent with the case reported by Kahveci, in which a 16-year-old patient with severe hypoxic-ischemic encephalopathy after out-of-hospital cardiac arrest showed marked neurological improvement following repeated mesenchymal stem cell therapy (4). That report, like ours, suggests that adjunctive MSC therapy may have a role in the post-resuscitation care of children who remain severely impaired after conventional intensive care. In recent years, MSC therapy has increasingly been proposed as a candidate adjunctive treatment to support neuroprotection and neurorestoration in children with brain injury. As is well known, the prognosis after traumatic brain injury is generally more favorable than after hypoxic brain injury following cardiac arrest; however, the risk of permanent disability and failure to recover is particularly high in patients with a GCS score of 8 or below. In this study, MSC therapy was offered to previously healthy children with GCS ≤ 8, after a joint decision between pediatric intensive care and pediatric neurology specialists, and only after detailed family counseling and informed consent. MSCs were administered at a dose of 1–2 × 10^6^ cells/kg per session via the IT or IV route, with the dose individualized according to body weight, age, and clinical condition. The number and frequency of sessions were also adjusted to each patient's status; published protocols have employed a range of regimens, including three sessions spaced 45 days apart or three consecutive daily administrations. In our cohort, five of six patients had comorbidities (neonatal hemorrhagic disease, laryngeal atresia, non-compaction cardiomyopathy, primary immunodeficiency, and a life-threatening arrhythmia), the indication for MSC therapy was hypoxic-ischemic injury after cardiac arrest in five patients and penetrating TBI in one, and the cell source was allogeneic bone marrow in four patients and allogeneic umbilical cord blood in two. The administration route was combined IT and IV in three patients, IV only in two, and IT only in one. In animal models, MSCs administered in the subacute period (3 days–2 weeks) have been shown to enhance neurogenesis and angiogenesis (15), and experimental studies have reported that MSC administration reduces inflammation, attenuates neuronal damage, and improves cognitive and motor function (16).

Although clinical evidence remains limited, several preliminary reports are encouraging. Administration of umbilical cord-derived MSCs has been associated with improvement in motor function in some studies (19), and improvements in GCS and functional recovery scores have been reported in children with TBI treated with autologous bone marrow-derived cell products (10). Other reports have suggested that different cell types may contribute to functional recovery by targeting damaged brain tissue (17), and preclinical studies in ischemic stroke models have shown that cell administration promotes recovery through neurogenesis and angiogenesis (28). As emphasized in the Introduction, however, BM-MNCs and UCB-MNCs are biologically distinct from purified, ISCT-criteria-compliant MSC products (8, 9), and observed differences in outcome across these studies likely reflect not only patient and protocol heterogeneity but also genuine differences in the cellular products themselves.

A Phase I clinical trial evaluating the safety and efficacy of cell therapy in children with severe TBI included 10 pediatric patients aged 5–14 years who received 6 × 10^6^ autologous bone marrow mononuclear cells per kilogram intravenously within 48 h of injury (11). At 6-month follow-up, 70% of patients had good outcomes and 30% had moderate to severe sequelae, based on GCS. Another Phase I study evaluated the IV administration of MSCs in children with brain damage from prenatal or postnatal hypoxia; no serious treatment-related adverse events were observed, although the need for longer-term follow-up was emphasized (10).

Routes of MSC administration in brain injury include IV infusion, IT injection, and intranasal (IN) delivery. IV infusion delivers cells via the systemic circulation, while IT injection delivers cells directly into the cerebrospinal fluid. IN administration has been explored as a minimally invasive alternative in preclinical models (29, 30). In children with severe TBI, IV MSC administration within the first 48 h after injury has been reported as safe (11). In children aged 5–14 years with a GCS of 5–8, IV autologous BM-MNC administered within the first 48 h after injury was associated with significant reductions in ICP, PELOD, and PICU duration (18), with reported reductions in mechanical ventilation duration of approximately three days and intensive care duration by 14%–23%. In the chronic phase (mean age 22 ± 27 months), allogeneic Wharton's jelly-derived MSCs (WJ-MSCs) administered at a dose of 1 × 10^6^ cells/kg via three different routes (IT, intramuscular, IV) every month for two months in six children with TBI were found to be safe at 12-month follow-up, with significant functional improvements (24). Functional gains have also been reported in conditions such as HIE and cerebral palsy with MSCs administered months after injury, although results vary across studies (20).

Because our patients had widespread cerebral edema and herniation findings at presentation, we elected to administer MSC therapy after the subacute period. It has been reported that intraventricular MSC administration in preterm intraventricular hemorrhage (IVH) models reduces ventricular expansion, while excessive doses may impair CSF flow (15). Increased ICP must be excluded before intrathecal MSC administration; otherwise, the risk of transtentorial herniation has been emphasized (18). In an experimental study, intracranial MSC injection of more than 10^6^ cells/kg was associated with an increased risk of intracranial hypertension (31). In pediatric TBI, an IV MSC dose of 6.8 × 10^6^ cells/kg was reported to be safe (11, 12), and an IV dose of 2 × 10^6^ MSC/kg was shown to reduce systemic inflammation (18). In a P9 rat model, intranasal administration of 0.5 × 10^6^ MSCs improved motor development (30), and in a preterm IVH model an intraventricular dose of 2 × 10^6^ MSCs was reported as safe (27).

Functional improvement has been reported with intrathecal administration of UC-MSCs in children with cerebral palsy (22), and improvements in motor scores have been observed after administration of allogeneic umbilical cord blood (UCB) (21). Drawing on these data, we administered 1–2 × 10^6^ cells/kg to our patients in 3–5 sessions, spaced 10 days to 2 weeks apart, after the subacute period and according to clinical condition. In two patients with significant herniation findings we selected the IV route; in one patient with chronic HIE sequelae we preferred the IT route to deliver cells more directly to the targeted brain. No procedural complications were observed.

Bone marrow-derived MSCs have the longest record of clinical use and are therefore the best characterized in terms of safety (7, 13). Adipose tissue-derived MSCs can be obtained using minimally invasive methods and are associated with a low procedural risk to the donor (6). Umbilical cord blood-derived MSCs are preferred in some studies because of their low immunogenicity (25). Autologous BM-MNC infusion has been reported to be safe in children with TBI (12). Functional improvement has been reported with intrathecal administration of UC-MSCs in children with cerebral palsy (22), and improvement in motor scores has been observed with allogeneic umbilical cord blood-derived products (23).

In the present study, within-cohort improvement in GCS and PCPC was observed after MSC therapy. The median GCS rose from 4 before MSC therapy to 11 at the end of the 6-month follow-up, and the median PCPC decreased from 5 at baseline to 4 at 1 month and 3 at 6 months. These within-cohort changes reached statistical significance, but—as emphasized throughout the manuscript—they cannot be interpreted as evidence of a causal effect of MSC therapy in this small uncontrolled cohort. The mechanistic rationale for any contribution of MSCs to recovery in this clinical setting rests on their paracrine and immunomodulatory effects rather than on direct neuronal replacement (14, 16, 17). Following hypoxic-ischemic or traumatic insult, secondary injury is propagated by neuroinflammation, oxidative stress, blood-brain barrier disruption, and progressive neuronal apoptosis (1, 2). Through the secretion of anti-inflammatory and trophic factors and the release of extracellular vesicles and exosomes, MSCs may modulate microglial activation, support endogenous neurogenesis and angiogenesis, restore blood-brain barrier integrity, and reduce reactive gliosis (13–17). We therefore view the role of MSCs in this clinical context as neuroprotective and pro-restorative rather than truly cell-replacing. Eligible patients had severe acquired brain injury and remained comatose despite optimal conventional neurocritical care; treatment was offered after detailed family counseling and was administered to those whose families consented and were able to access this non-reimbursed adjunctive therapy (see Limitations regarding selection bias). Because patients were followed in their home provinces, long-term motor outcomes could not be systematically captured; PCPC at 1 and 6 months therefore served as our primary functional outcome measure.

### Limitations

This study has several important limitations that constrain interpretation.

First, this is a retrospective, uncontrolled case series of only six patients. Without a contemporaneous control group, observed neurological improvement cannot be distinguished from natural recovery, the effects of high-quality conventional neurocritical care, the impact of early rehabilitation, or regression to the mean. Statistical tests describe within-cohort change over time and cannot be interpreted as evidence of a causal effect of MSC therapy.

Second, the cohort is clinically and biologically heterogeneous. Five patients had hypoxic-ischemic injury and one had penetrating TBI; patients differed in age, comorbidity profile, severity, MSC source (bone marrow vs. umbilical cord blood), administration route (IV, IT, or combined), and number of sessions. This heterogeneity precludes generalization to either the hypoxic-ischemic or the traumatic population specifically.

Third, the cohort is subject to selection bias inherent to the Turkish healthcare context. MSC therapy is not reimbursed by national health insurance for any pediatric indication and is not part of standard PICU care. Only patients whose families could access the treatment financially, and who consented after detailed counseling about its experimental nature, were included. This non-clinical selection factor limits external generalizability and should be borne in mind when interpreting our results. Future studies conducted within prospective trial frameworks that cover treatment costs would be needed to mitigate this bias and broaden access.

Fourth, long-term follow-up was conducted in patients' home provinces by local pediatric neurology services, with variable access to advanced neuroimaging and electrophysiology. Standardized longitudinal imaging or EEG correlation with clinical outcomes was therefore not feasible. While qualitative imaging features are described, no quantitative imaging biomarker analysis could be performed.

Fifth, although safety monitoring was systematic and no adverse events were detected, the sample size is far too small to support meaningful safety inference, particularly for the IT route. Larger prospective cohorts with predefined adverse event capture are required for safety conclusions.

Finally, although the biological products were characterized in accordance with ISCT criteria, the dose, schedule, route, and number of sessions varied across patients and were individualized to clinical context. This precludes inference about an optimal protocol from these data (27).

## Conclusion

Traumatic and non-traumatic brain injury substantially impair the short- and long-term quality of life of affected children and place a considerable burden on families and health systems (4, 31). Treatments capable of supporting long-term recovery in both traumatic and non-traumatic acquired brain injury would have an important impact in this area, and adjunctive MSC therapy is one approach with therapeutic and preliminary clinical rationale (4). In this small retrospective case series of six children, adjunctive MSC therapy in addition to standardized conventional neurocritical care was feasible and well tolerated, and within-cohort neurological improvement was observed during follow-up. These observations are preliminary and hypothesis-generating; they should not be interpreted as evidence of efficacy. Multicenter, adequately powered, prospective, randomized, and controlled clinical trials with standardized cell products, defined administration protocols, and predefined imaging and functional endpoints are needed before adjunctive MSC therapy can be reliably integrated into pediatric neurocritical care.

## Data Availability

The original contributions presented in the study are included in the article/Supplementary Material, further inquiries can be directed to the corresponding author.

## References

[1] Kochanek PM Tasker RC Carney N Totten AM Adelson PD Selden NR. Guidelines for the management of pediatric severe traumatic brain injury, third edition. Pediatr Crit Care Med. (2019) 20(3 Suppl 1):S1–82. 10.1097/PCC.0000000000001735 30829890

[2] Loane DJ Kumar A. Microglia in the TBI brain: the good, the bad, and the dysregulated. Exp Neurol. (2016) 275(Pt 3):316–27. 10.1016/j.expneurol.2015.08.018 26342753 PMC4689601

[3] Silverstein FS Lai MC. Hypoxic-ischemic encephalopathy and cerebral palsy. In: Gleason CA Juul SE, editors. Avery’s Diseases of the Newborn. 11th ed. Philadelphia: Elsevier (2020). pp. 1056–72.

[4] Kahveci F. Unwitnessed out-of-hospital cardiac arrests in children: can mesenchymal stem cells work at post-resuscitation care? Turk Arch Pediatr. (2025) 60(5):562–3. 10.5152/TurkArchPediatr.2025.24275 40960382 PMC12432205

[5] Squillaro T Peluso G Galderisi U. Clinical trials with mesenchymal stem cells: an update. Cell Transplant. (2016) 25(5):829–48. 10.3727/096368915X689622 26423725

[6] Wei X Yang X Han ZP Qu FF Shao L Shi YF. Mesenchymal stem cells: a new trend for cell therapy. Acta Pharmacol Sin. (2013) 34(6):747–54. 10.1038/aps.2013.50 23736003 PMC4002895

[7] Pittenger MF Mackay AM Beck SC Jaiswal RK Douglas R Mosca JD. Multilineage potential of adult human mesenchymal stem cells. Science. (1999) 284(5411):143–7. 10.1126/science.284.5411.143 10102814

[8] Dominici M Le Blanc K Mueller I Slaper-Cortenbach I Marini FC Krause DS. Minimal criteria for defining multipotent mesenchymal stromal cells. The international society for cellular therapy position statement. Cytotherapy. (2006) 8(4):315–7. 10.1080/14653240600855905 16923606

[9] Ullah I Subbarao RB Rho GJ. Human mesenchymal stem cells: current trends and future prospective. Biosci Rep. (2015) 35(2):e00191. 10.1042/BSR20150025 25797907 PMC4413017

[10] Lin W-Y Wu K-H Chen C-Y Guo B-C Chang Y-J Lee T-A. Stem cell therapy in children with traumatic brain injury. Int J Mol Sci. (2023) 24(19):14706. 10.3390/ijms241914706 37834152 PMC10573043

[11] Cox CS Jr Baumgartner JE Harting MT Worth LL Walker PA Shah SK. Autologous bone marrow mononuclear cell therapy for severe traumatic brain injury in children. Neurosurgery. (2011) 68(3):588–600. 10.1227/NEU.0b013e318207734c 21192274

[12] Cox CS Jr Notrica DM Juranek J Miller JH Triolo F Kosmach S. Autologous bone marrow mononuclear cells to treat severe pediatric traumatic brain injury. Brain. (2024) 147(5):1914–25. 10.1093/brain/awae005 38181433 PMC11068104

[13] Caplan AI Correa D. The MSC: an injury drugstore. Cell Stem Cell. (2011) 9(1):11–5. 10.1016/j.stem.2011.06.008 21726829 PMC3144500

[14] Teixeira FG Carvalho MM Sousa N Salgado AJ. Mesenchymal stem cells secretome: a new paradigm for CNS regeneration? Cell Mol Life Sci. (2013) 70(20):3871–82. 10.1007/s00018-013-1290-8 23456256 PMC11113366

[15] Chen J Li Y Wang L Lu M Zhang X Chopp M. Therapeutic benefit of intracerebral transplantation of bone marrow stromal cells after cerebral ischemia in rats. J Neurol Sci. (2001) 189(1–2):49–57. 10.1016/S0022-510X(01)00557-3 11535233

[16] Zhang Y Zheng Z Sun J. Application of mesenchymal stem cells in traumatic brain injury: mechanisms, results, and problems. Histol Histopathol. (2024) 39(9):1109–31. 10.14670/HH-18-716 38353136

[17] Zhang Y Chopp M Meng Y Katakowski M Xin H Mahmood A. Effect of exosomes derived from mesenchymal stromal cells on recovery after traumatic brain injury. J Neurosurg. (2015) 122(4):856–67. 10.3171/2014.11.JNS14770 25594326 PMC4382456

[18] Liao GP Harting MT Hetz RA Walker PA Shah SK Corkins CJ. Autologous bone marrow mononuclear cells reduce therapeutic intensity for severe traumatic brain injury in children. Pediatr Crit Care Med. (2015) 16(3):245–55. 10.1097/PCC.0000000000000324 25581630 PMC4351120

[19] Wang S Cheng H Dai G Wang X Hua R Liu X. Umbilical cord mesenchymal stem cell transplantation significantly improves neurological function in patients with traumatic brain injury. Brain Res. (2013) 1532:76–84. 10.1016/j.brainres.2013.08.001 23942181

[20] Xue L Du R Bi N Xiao Q Sun Y Niu R. Placental chorionic plate-derived mesenchymal stem cells for neonatal hypoxic-ischemic encephalopathy. Neural Regen Res. (2024) 19(9):2027–35. 10.4103/1673-5374.390952 38227532 PMC11040304

[21] Lee TK Lu CY Tsai ST Tseng PH Lin YC Lin SZ. Safety and feasibility of allogeneic umbilical cord blood monocytes in patients with acute cerebral stroke: a phase I clinical trial. Cell Transplant. (2021) 30:9636897211067447. 10.1177/09636897211067447 34939863 PMC8728774

[22] Akhlaghpasand M Hosseinpoor M Hajikarimloo B Hajarizadeh A Golmohammadi M Tavanaei R. Repeated intrathecal autologous mesenchymal stem cells for spastic cerebral palsy. J Neurorestoratology. (2025) 13:100207. 10.1016/j.jnrt.2025.100207

[23] Feng M Lu A Gao H Qian C Zhang J Lin T. Safety of allogeneic umbilical cord blood stem cells therapy in patients with severe cerebral palsy: a retrospective study. Stem Cells Int. (2015) 2015:325652. 10.1155/2015/325652 26236347 PMC4510256

[24] Kabatas S Civelek E Boyalı O Sezen GB Ozdemir O Bahar-Ozdemir Y. Safety and efficiency of wharton’s jelly-derived mesenchymal stem cell administration in patients with traumatic brain injury. World J Stem Cells. (2024) 16(6):641–55. 10.4252/wjsc.v16.i6.641 38948099 PMC11212551

[25] Kim DW Staples M Shinozuka K Pantcheva P Kang SD Borlongan CV. Wharton’s jelly-derived mesenchymal stem cells: phenotypic characterization and optimizing their therapeutic potential for clinical applications. Int J Mol Sci. (2013) 14(6):11692–712. 10.3390/ijms140611692 23727936 PMC3709752

[26] Fischer UM Harting MT Jimenez F Monzon-Posadas WO Xue H Savitz SI. Pulmonary passage is a major obstacle for intravenous stem cell delivery. Stem Cells Dev. (2009) 18(5):683–92. 10.1089/scd.2008.0253 19099374 PMC3190292

[27] Park WS Sung SI Ahn SY Sung DK Im GH Yoo HS. Optimal timing of mesenchymal stem cell-mediated treatment for neonatal intraventricular hemorrhage. Cell Transplant. (2016) 25(6):1131–44. 10.3727/096368915X689640 26440762

[28] Kalladka D Muir KW. Stem cell therapy in stroke: designing clinical trials. Neurochem Int. (2011) 59(3):367–70. 10.1016/j.neuint.2011.03.016 21453739

[29] Danielyan L Schäfer R Von Ameln-Mayerhofer A Buadze M Geisler J Klopfer T. Intranasal delivery of cells to the brain. Eur J Cell Biol. (2009) 88(6):315–24. 10.1016/j.ejcb.2009.02.001 19324456

[30] Donega V Van Velthoven CTJ Nijboer CH Van Bel F Kas MJH Kavelaars A. Intranasal mesenchymal stem cell treatment for neonatal brain damage. PLoS One. (2013) 8(1):e51253. 10.1371/journal.pone.0051253 23300948 PMC3536775

[31] Chen KH Lin KC Wallace CG Li YC Shao PL Chiang JY. iPSC-derived mesenchymal stem cell therapy reduces brain infarct volume in rats. Am J Transl Res. (2019) 11(9):6232–48. PMCID: PMC6789217 31632590 PMC6789217

